# Multimodal OCT–OCTA Classification of Dry AMD, Active MNV, and Inactive MNV Using Frozen Vision Transformer Features: A Patient-Level Nested Cross-Validation Study

**DOI:** 10.3390/jcm15218647

**Published:** 2026-11-06

**Authors:** İlknur Tuncer Fırat, Murat Fırat, Emrah Öztürk, Taner Tuncer

**Affiliations:** 1 Department of Ophthalmology, Faculty of Medicine, Turgut Özal Medical Center, İnönü University, Malatya 44280, Türkiye; emrah.ozturk@inonu.edu.tr; 2 Department of Ophthalmology, Faculty of Medicine, Malatya Turgut Özal University, Malatya 44210, Türkiye; murat.firat@ozal.edu.tr; 3 Department of Computer Engineering, Faculty of Engineering, Fırat University, Elazig 23119, Türkiye; ttuncer@firat.edu.tr

**Keywords:** age-related macular degeneration, macular neovascularization, optical coherence tomography, optical coherence tomography angiography, vision transformer, multimodal imaging

## Abstract

**Background/Objectives**: Distinguishing dry age-related macular degeneration (AMD) from active and inactive macular neovascularization (MNV) may benefit from integrated interpretation of optical coherence tomography (OCT) and OCT angiography (OCTA). However, the added value of OCTA under artifact-prone conditions remains uncertain. We compared OCT-only, direct OCTA fusion, and OCT–OCTA multimodal classification using frozen Vision Transformer features. **Methods**: This retrospective study included 368 eyes from 200 patients. OCT B-scans and outer retina and choriocapillaris OCTA images were analyzed. A pretrained Vision Transformer B/32 (ViT-B/32) generated modality-specific embeddings. Class-weighted linear support vector machine (SVM) models were developed using patient-grouped repeated nested cross-validation (10 repetitions; five outer and four inner folds). The primary gradable cohort comprised 320 eyes from 185 patients; severe OCTA artifacts and non-exudative MNV were assessed separately in predefined challenge cohorts. **Results**: Direct multimodal fusion achieved an accuracy of 0.753, balanced accuracy of 0.679, macro-F1 of 0.685, and macro area under the receiver operating characteristic curve (AUROC) of 0.887. It significantly outperformed direct OCTA fusion across five prespecified metrics; differences from OCT alone were not statistically significant. Class-specific sensitivities were 0.916 for dry AMD, 0.709 for active MNV, and 0.412 for inactive MNV. Performance estimates were similar in the real-world cohort (accuracy, 0.758; macro-AUROC, 0.886). Whole-modality occlusion suggested predominant OCT reliance for dry AMD and greater outer retina OCTA reliance for active and inactive MNV. **Conclusions**: Frozen Vision Transformer-derived multimodal features supported three-class OCT–OCTA classification under patient-level validation. Structural OCT provided most of the discriminative information. OCTA influenced model predictions in occlusion analyses, but its incremental classification benefit was not established. External multicenter validation is required before clinical use.

## 1. Introduction

Age-related macular degeneration (AMD) is a leading cause of irreversible central vision loss among older adults [1,2]. The disease is broadly classified into non-neovascular, or dry, AMD and neovascular AMD (nAMD), which is characterized by the development of macular neovascularization (MNV) [2,3]. Dry AMD is associated with drusen, retinal pigment epithelium alterations, and, in advanced stages, atrophy involving the outer retina and retinal pigment epithelium [2]. In nAMD, abnormal vascular complexes may cause exudation, hemorrhage, fibrosis, and rapid visual loss [3]. Active MNV is generally defined by exudative findings such as intraretinal fluid, subretinal fluid, or hemorrhage, whereas inactive MNV refers to previously diagnosed nAMD without evidence of current activity at the imaging visit [3,4]. This distinction is important for initiating and continuing anti-vascular endothelial growth factor (anti-VEGF) treatment and determining treatment intervals [1,4].

Optical coherence tomography (OCT) is the principal imaging modality used for the diagnosis and monitoring of AMD [5,6]. It enables the assessment of structural findings such as drusen, pigment epithelial detachment, subretinal hyperreflective material, outer retinal damage, atrophy, intraretinal fluid, and subretinal fluid [5]. The detection of intraretinal and subretinal fluid is particularly important for evaluating nAMD activity and guiding retreatment decisions [1,5]. The ability to obtain comparable images across consecutive visits also facilitates monitoring of the anatomical response to anti-VEGF therapy [5,6].

Optical coherence tomography angiography (OCTA) uses flow-related signal changes between repeated scans to visualize retinal and choroidal vasculature without dye injection [7]. Layer-specific en face images permit evaluation of the location, extent, and vascular morphology of MNV complexes within the outer retinal and choriocapillaris slabs [8]. Features such as fine peripheral branching, anastomoses, and a perilesional dark halo may provide information regarding neovascular activity [8]. However, because OCTA does not directly demonstrate vascular permeability or leakage, treatment decisions cannot be based on OCTA findings alone [7].

Combined evaluation of OCT and OCTA may help distinguish the presence of an MNV complex from current exudative activity [8]. Nevertheless, OCTA images are susceptible to motion, projection, segmentation, shadowing, defocus, and decentration artifacts [9,10]. Slow flow below the detection threshold and inaccurate slab boundary placement may also obscure or misrepresent MNV [10]. OCTA findings should therefore be interpreted together with matched structural OCT images in clinical practice [7,8].

Artificial intelligence methods are increasingly being applied to the automated analysis of structural and vascular features in OCT and OCTA images [11,12,13]. Leingang et al. demonstrated automated classification of early or intermediate, atrophic, and neovascular AMD using real-world OCT volumes [11]. Schranz et al. used artificial intelligence–derived OCT fluid measurements to examine associations between exudative activity and OCTA-derived MNV characteristics [12]. Multimodal models combining OCT and OCTA have also been evaluated for multiclass classification across macular and retinal diseases [13].

Despite these advances, direct comparisons of OCT-only, OCTA-only, and combined OCT–OCTA approaches for differentiating dry AMD, active MNV, and inactive MNV within the same patient-level validation framework remain limited. Moreover, the effects of common OCTA artifacts and structural–vascular discordance associated with non-exudative MNV on model predictions have not been adequately characterized [7,8,9,10]. In this study, image features were extracted from structural OCT and two OCTA slabs using a pretrained Vision Transformer with frozen weights. OCT-only classification, direct OCTA fusion, and direct OCT–OCTA multimodal fusion were compared using patient-grouped repeated nested cross-validation. We additionally evaluated class-specific performance, calibration, predictive uncertainty, severe OCTA artifacts, non-exudative MNV, and model sensitivity to modality-specific perturbations.

## 2. Materials and Methods

### 2.1. Study Design and Ethics

This single-center retrospective imaging study included the clinical records and matched images of patients with age-related macular degeneration (AMD) who were evaluated at the retina unit of İnönü University Turgut Özal Medical Center between January 2021 and March 2025. The study was approved by the İnönü University Health Sciences Scientific Research Ethics Committee (Approval No. 2025/7558; 24 June 2025) and was conducted in accordance with the Declaration of Helsinki. The requirement for written informed consent was waived because of the retrospective design and the use of anonymized data.

Patients aged 50 years or older were eligible when a same-day matched image triplet consisting of an optical coherence tomography (OCT) B-scan, an outer retina OCT angiography (OCTA) image, and a choriocapillaris OCTA image was available. One or both eyes from the same patient could be included. Eyes with macular neovascularization (MNV) secondary to conditions other than AMD, including pathologic myopia, central serous chorioretinopathy, and angioid streaks, or with other macular disorders that could interfere with classification were excluded. Eyes with clinically significant media opacities documented in the medical records were excluded, whereas milder opacities were not grounds for exclusion, and any associated image degradation was considered during OCTA artifact grading.

The complete dataset comprised 368 eyes from 200 patients: 183 with dry AMD, 105 with active MNV, and 80 with inactive MNV. Each eye contributed one matched OCT–OCTA image triplet. When multiple imaging visits were available for an eye, the earliest examination providing an adequately interpretable same-day OCT/OCTA image set was preferentially selected.

### 2.2. Diagnostic Reference Standard and Image Acquisition

Diagnostic classification was based primarily on structural OCT findings and clinical history. Active MNV was defined by the presence of subretinal and/or intraretinal fluid on OCT in an eye with neovascular AMD. Inactive MNV was assigned to previously diagnosed neovascular AMD without subretinal or intraretinal fluid at the imaging visit. Dry AMD was defined by non-neovascular AMD findings without previous exudative MNV or current retinal fluid. OCTA findings were reviewed together with OCT but did not replace the OCT-based activity definition. Eyes without retinal fluid or previous exudative MNV but showing an OCTA-detected neovascular complex were designated as non-exudative MNV. These eyes were excluded from model development and evaluated separately as a predefined challenge cohort.

All images were acquired using the same swept-source OCT device (DRI OCT Triton; Topcon Corporation, Tokyo, Japan). According to the manufacturer’s specifications, the device provides an axial optical resolution of 8 µm (digital resolution: 2.6 µm) and a lateral resolution of 20 µm. A fovea-centered 6 mm radial OCT scan and a 6 × 6 mm OCTA scan were obtained on the same day. The horizontal OCT B-scan was used as the structural input, and the automatically segmented en face outer retina, and choriocapillaris slabs were used as the two OCTA inputs.

Reference diagnoses were established by three ophthalmologists (M.F., E.Ö. and İ.T.F.) through joint review of the complete OCT scan set, available clinical records, and corresponding OCTA images. Device-acquired color fundus photographs were additionally reviewed when diagnostic uncertainty arose, although the number of such cases was not recorded. Disagreements were resolved according to the majority opinion of the three ophthalmologists. Because individual assessments were not independently recorded, formal intergrader agreement could not be calculated. Importantly, the presence or absence of intraretinal and subretinal fluid was determined from the complete OCT scan set, whereas only a single horizontal OCT B-scan was provided as the structural input to the classifier.

The active MNV group included both newly diagnosed eyes and previously treated eyes with current exudative activity. All eyes classified as inactive MNV were receiving anti-VEGF treatment. Although clinical records were available during diagnostic assessment, detailed treatment variables, including injection frequency, cumulative treatment burden, and time since the most recent injection, were not systematically collected in the study dataset.

### 2.3. Image Quality and Analysis Cohorts

The device-derived OCT image-quality score was recorded. OCTA images were evaluated for motion or banding, shadowing or masking, segmentation error, projection artifact, blur or defocus, unmasking or window defects, and decentration. Artifact severity was graded from 0 to 3 according to its effect on lesion discernibility, with grade 3 indicating that assessment was very difficult or clinically unreliable. Detailed artifact definitions and grading criteria are provided in Supplementary Method S1, and representative examples are shown in Supplementary Figure S1.

The primary gradable cohort excluded non-exudative MNV and grade-3 artifacts and comprised 320 eyes from 185 patients: 166 with dry AMD, 86 with active MNV, and 68 with inactive MNV. The real-world cohort included all artifact-severity grades but excluded non-exudative MNV, yielding 355 eyes from 198 patients. The predefined challenge cohorts consisted of 35 eyes from 27 patients with grade-3 artifacts and 13 eyes from 11 patients with non-exudative MNV. Model development and hyperparameter selection were restricted to the primary gradable cohort. All 368 included eyes had complete data for age, sex, OCT quality score, diagnostic label, and OCTA artifact severity, together with the three required imaging inputs. No missing-value imputation or available-case analyses were required for these variables. The primary gradable, grade-3 artifact, and non-exudative MNV subgroups were mutually exclusive at the eye level. Because patients could contribute different eyes to different subgroups, patient counts were not additive (Supplementary Figure S2).

### 2.4. Image Preprocessing and Feature Extraction

Device-generated colored scan guides were deterministically masked before grayscale conversion. Images were resized to 224 × 224 pixels and normalized using ImageNet parameters; no image augmentation was applied. The inspected original image archive showed variable exported image dimensions (OCT: 1037–1067 × 536–585 pixels; OCTA: predominantly 1606–1657 × 678–713 pixels). A uniform cropping procedure was applied across all diagnostic groups to remove peripheral device-generated annotations before resizing the processed images to 224 × 224 pixels using bicubic interpolation. Full preprocessing specifications and the predefined masked-versus-unmasked sensitivity analysis are described in Supplementary Methods S2 and S6.

Features were extracted independently from each modality using an ImageNet-1K–pretrained Vision Transformer B/32 (ViT-B/32), based on the architecture described by Dosovitskiy et al. [14] and implemented using PyTorch 2.5.1 and torchvision 0.20.1 (torchvision.models.vit_b_32, ViT_B_32_Weights.IMAGENET1K_V1). The encoder weights remained frozen, and no end-to-end fine-tuning was performed. A 768-dimensional embedding was obtained from each OCT, outer retina OCTA, and choriocapillaris OCTA image. ViT-B/32 was selected for its available pretrained weights, self-attention-based representation of spatial relationships, and ability to provide standardized features across all three imaging modalities, rather than on demonstrated superiority over CNN architectures.

Three feature configurations were evaluated: OCT only, direct OCTA fusion combining the two OCTA embeddings, and direct multimodal fusion combining all three embeddings. Direct multimodal fusion was prespecified as the primary input configuration. Image-quality, artifact, age, and sex variables were not provided to the classifiers. Additional preprocessing, embedding, and feature-construction details are provided in Supplementary Method S2.

### 2.5. Model Development and Validation

A class-weighted linear support vector machine (SVM) was prespecified as the primary classifier. Within each training fold, features were standardized and reduced using principal component analysis with up to 128 components. This frozen-encoder, PCA, and linear-classifier approach was chosen to limit model complexity in the relatively small cohort rather than train an end-to-end deep learning model. Hyperparameters were selected by inner cross-validation using macro-F1, and the final SVM scores were converted to class probabilities using sigmoid calibration. Elastic-net multinomial logistic regression was evaluated as a predefined secondary classifier. Exact model specifications, candidate hyperparameters, selection procedures, and calibration methods are provided in Supplementary Methods S4 and S5.

Model development and evaluation used patient-grouped repeated nested cross-validation with 10 repetitions, five outer folds, and four inner folds. Both eyes from the same patient were retained in the same fold. Outer-fold assignments were generated across all 200 patients, including those represented only in the challenge cohorts, and all eyes from each outer-test patient were excluded from model training, hyperparameter selection, preprocessing fitting, and probability calibration, even when the fellow eye qualified for the primary gradable cohort. All data-dependent transformations, including feature standardization and principal component analysis, as well as hyperparameter selection, model fitting, and probability calibration, were performed exclusively within the corresponding training data. Patient-level fold construction and integrity checks are described in Supplementary Method S3.

Each eye received one outer out-of-fold probability vector per repetition. Final eye-level probabilities were calculated by averaging the 10 repeated out-of-fold predictions, and the class with the highest mean probability was selected. For the challenge cohorts, predictions were generated only when the corresponding patient served in the outer-test fold, thereby maintaining patient-level separation from the training data.

### 2.6. Performance Assessment and Statistical Analysis

Overall performance was evaluated using accuracy, balanced accuracy, macro-F1, Matthews correlation coefficient, Cohen’s kappa, macro one-versus-rest area under the receiver operating characteristic curve, and macro average precision. Probability-based performance was assessed using log loss, multiclass Brier score, and macro expected calibration error. Class-specific sensitivity, specificity, precision, F1 score, area under the receiver operating characteristic curve, and average precision were also calculated.

Confidence intervals were estimated using 2000 patient-clustered bootstrap replicates, retaining all eyes from each sampled patient together. Paired comparisons of the primary multimodal SVM with the OCT-only and direct-OCTA SVM models used identical bootstrap samples. Two-sided bootstrap *p* values were adjusted using the Holm method within each performance metric.

Prespecified secondary analyses evaluated preprocessing sensitivity, misclassification pathways, predictive uncertainty, and modality reliance. Whole-modality occlusion replaced each imaging input with its image-specific median intensity, whereas local patch-occlusion analysis was performed for representative errors. These analyses quantify model sensitivity to predefined perturbations and should not be interpreted as demonstrating causal anatomical importance. Detailed definitions and procedures are provided in Supplementary Methods S6–S8.

Because the non-exudative MNV cohort contained only one reference class, multiclass discrimination metrics were not estimable; the dry-AMD assignment rate and mean dry-AMD probability were reported instead. Software versions and reproducibility controls are provided in Supplementary Method S9.

**Figure 1:**
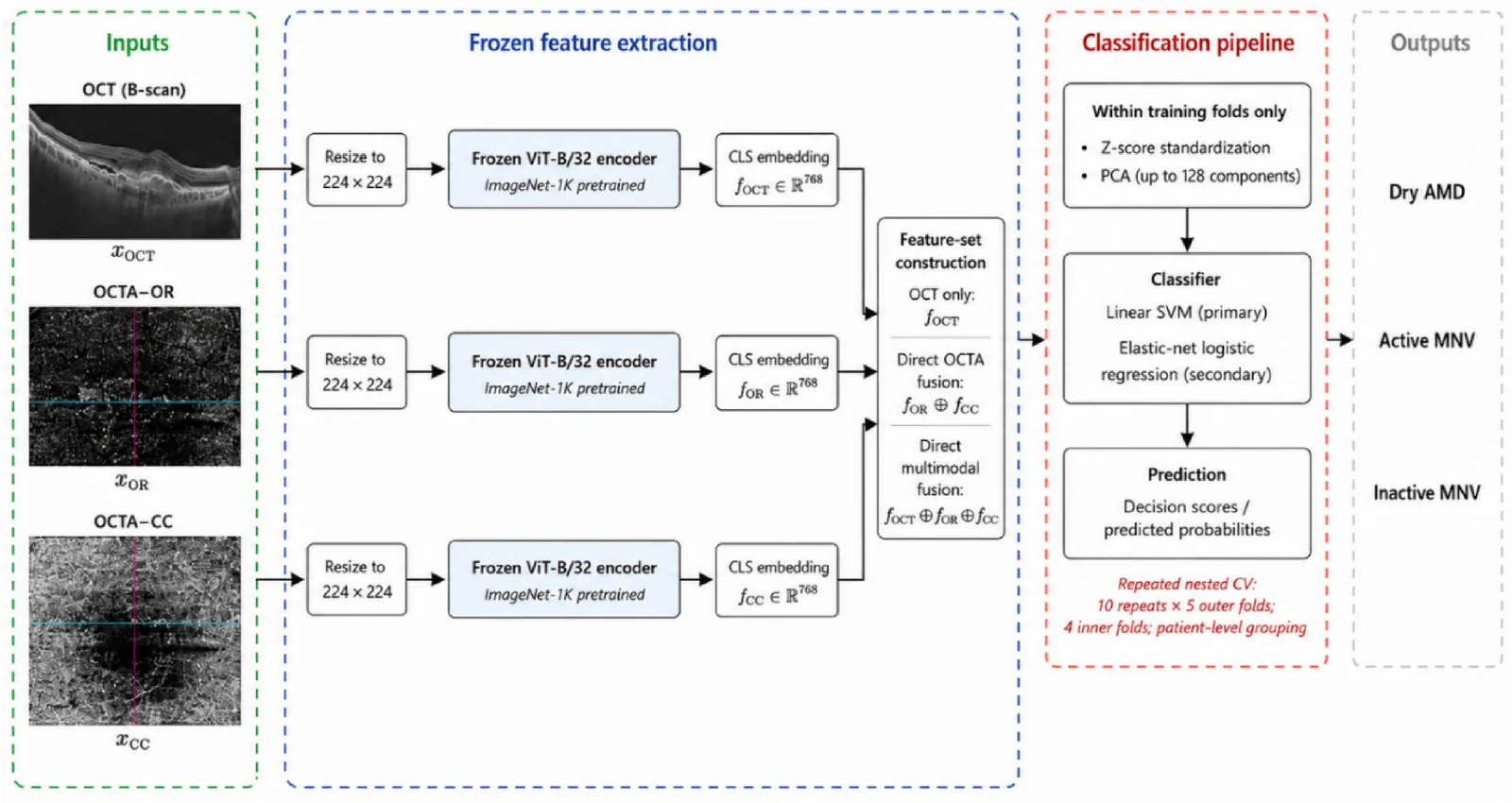
Overview of the multimodal OCT–OCTA classification pipeline. OCT B-scan, outer retina OCTA, and choriocapillaris OCTA images were encoded using a frozen ImageNet-pretrained ViT-B/32. OCT-only, direct OCTA fusion, and direct multimodal fusion were evaluated using patient-grouped repeated nested cross-validation. Standardization, principal component analysis, model selection, and probability calibration were performed within the training folds only.

The overall analysis workflow is summarized in Figure 1.

## 3. Results

### 3.1. Study Cohort and Image Characteristics

The study included 368 eyes from 200 patients. The mean participant age was 70.5 ± 7.5 years, and 81 participants (40.5%) were female. The eye-level dataset comprised 183 eyes with dry age-related macular degeneration (AMD), 105 with active macular neovascularization (MNV), and 80 with inactive MNV. OCTA artifacts were more frequent in the MNV groups, although most images remained gradable. After excluding non-exudative MNV and grade-3 artifacts, the primary gradable cohort comprised 320 eyes from 185 patients (Table 1). Detailed distributions of artifact severity and type are provided in Supplementary Table S1.

### 3.2. Overall Performance of the Primary Models

Direct multimodal fusion achieved an accuracy of 0.753, balanced accuracy of 0.679, macro-F1 of 0.685, and macro-AUROC of 0.887 in the primary cohort (Table 2). Hyperparameter-selection frequencies and inner-cross-validation profiles are summarized in Supplementary Table S2 and Supplementary Figure S3. Direct multimodal fusion significantly outperformed direct OCTA fusion in five paired metrics, whereas differences from OCT alone were not statistically significant (Supplementary Table S3).

In secondary analyses, the direct multimodal elastic-net model produced higher point estimates for several threshold-dependent metrics, with discrimination similar to that of the primary linear support vector machine (SVM). Because the linear SVM was prespecified as the primary classifier, it was retained for all principal and challenge-cohort analyses. Performance estimates for all predefined classifier and input configurations are provided in Supplementary Table S4.

### 3.3. Class-Specific Discrimination

**Figure 2:**
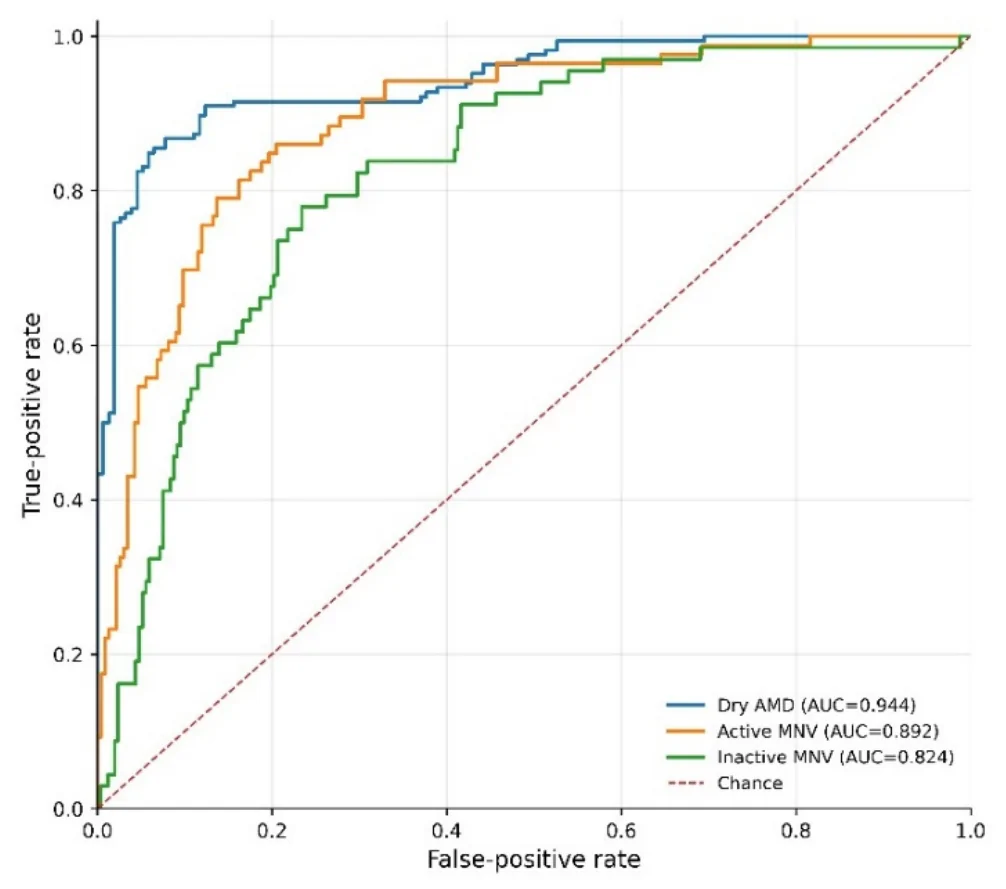
One-versus-rest receiver operating characteristic curves for the primary multimodal linear SVM in the primary cohort. AUROC was 0.944 for dry AMD, 0.892 for active MNV, and 0.824 for inactive MNV.

**Figure 3:**
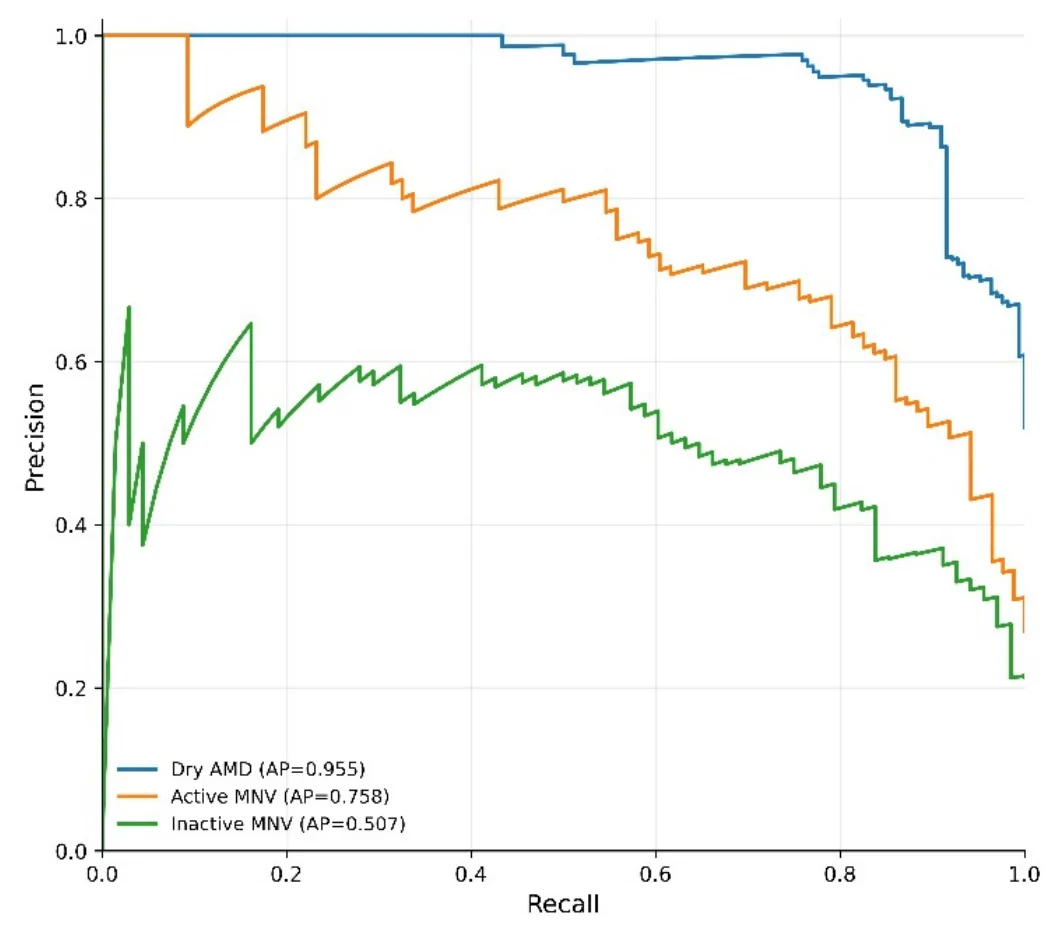
One-versus-rest precision–recall curves for the primary multimodal linear SVM in the primary cohort. AP was 0.955 for dry AMD, 0.758 for active MNV, and 0.507 for inactive MNV.

Dry AMD showed the highest discrimination, with an AUROC of 0.944 and average precision (AP) of 0.955. Corresponding values were 0.892 and 0.758 for active MNV and 0.824 and 0.507 for inactive MNV (Figure 2 and Figure 3). Thresholded classification showed the same pattern: sensitivity was 0.916 for dry AMD, 0.709 for active MNV, and 0.412 for inactive MNV (Table 3).

### 3.4. Confusion Patterns and Calibration

**Figure 4:**
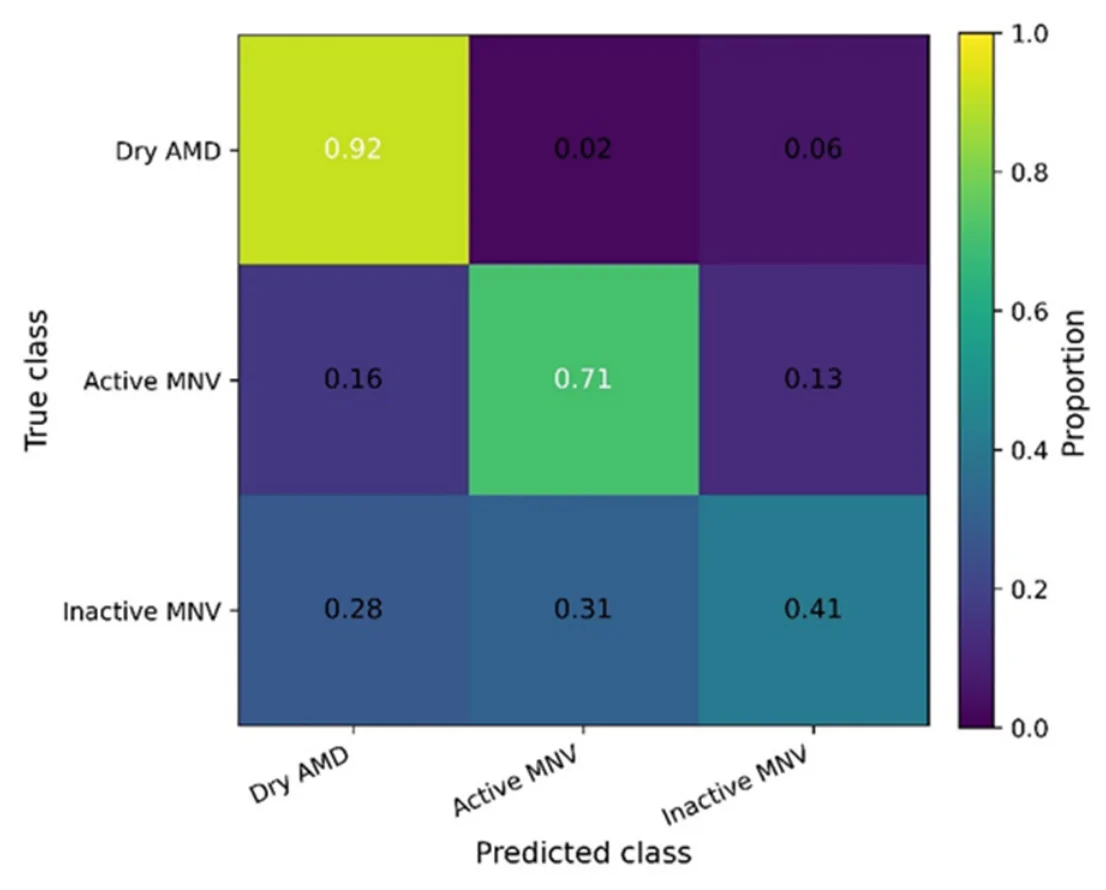
Row-normalized confusion matrix for the primary multimodal linear SVM in the primary cohort. Cells show the proportion of each reference class assigned to each predicted class.

**Figure 5:**
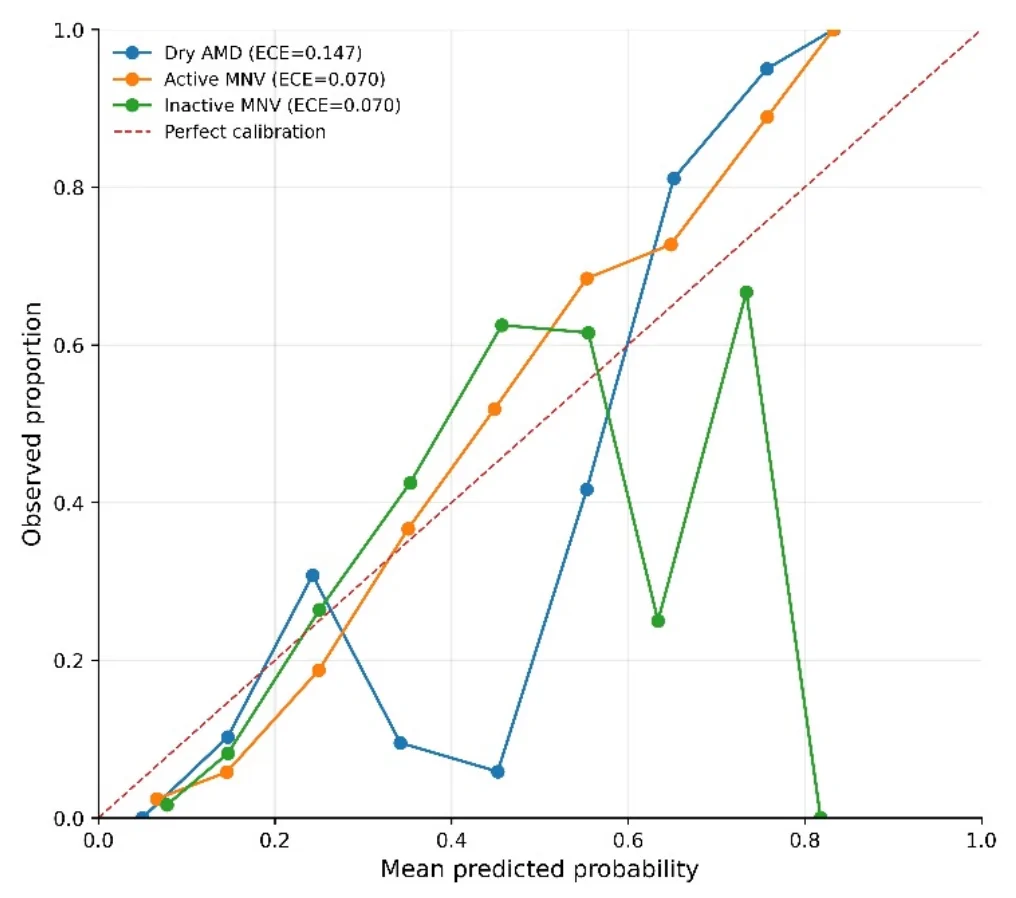
One-versus-rest calibration curves for the primary multimodal linear SVM. Curves were constructed using 10 equal-width probability bins. ECE was 0.147 for dry AMD and 0.070 for both active and inactive MNV.

The model correctly classified 91.6% of dry AMD, 70.9% of active MNV, and 41.2% of inactive MNV eyes. Inactive MNV was frequently assigned to either active MNV or dry AMD (Figure 4). Calibration varied by class. Expected calibration error (ECE) was 0.147 for dry AMD and 0.070 for both active and inactive MNV, yielding a macro-ECE of 0.096. Fluctuations at higher inactive-MNV probabilities should be interpreted cautiously because some calibration bins contained few observations (Figure 5).

### 3.5. Robustness and Challenge-Cohort Performance

Performance estimates in the real-world cohort, which included eyes with grade-3 OCTA artifacts, were similar to those in the primary gradable cohort (Table 4). Point estimates were also favorable in the grade-3 artifact cohort; however, this subgroup comprised only 35 eyes from 27 patients and had wide confidence intervals. These estimates should therefore be interpreted cautiously and should not be considered evidence that severe artifacts improve classification.

The non-exudative MNV challenge cohort comprised 13 eyes from 11 patients. Eight eyes (61.5%; 95% CI, 30.8–91.7%) were assigned to dry AMD, and the mean predicted probability of dry AMD was 0.488 (95% CI, 0.404–0.570). Because this cohort contained only one reference category, multiclass performance metrics were not estimable.

Omitting chromatic scan-guide masking produced only small changes in the primary and real-world cohorts. Differences favoring masking in the grade-3 subgroup were exploratory (Supplementary Table S5 and Supplementary Figure S4).

### 3.6. Misclassification and Predictive Uncertainty

Seventy-nine of 320 primary-cohort eyes were misclassified. Forty errors arose from inactive MNV, most often through assignment to active MNV or dry AMD. Assigned-class probabilities were generally close to 0.50, suggesting that many errors occurred near the model’s decision boundaries (Table 5).

Whole-modality occlusion showed class-dependent reliance on the three imaging inputs (Supplementary Table S6). Complete predictive entropy and across-repeat variability results are provided in Supplementary Table S7. Local patch-occlusion maps for representative errors are shown in Supplementary Figure S5.

**Figure 6:**
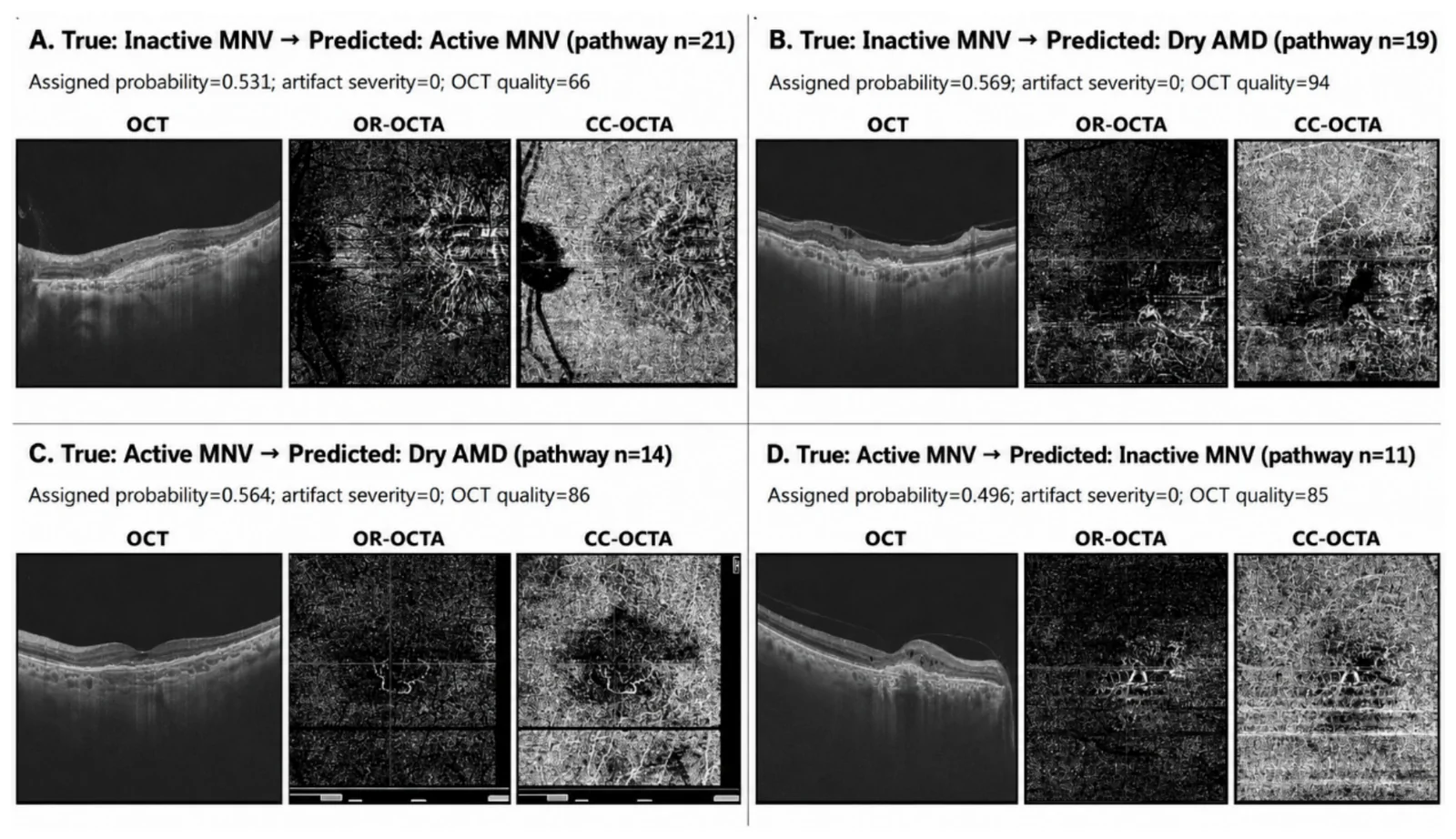
Representative examples from the four most frequent misclassification pathways: (**A**) inactive MNV classified as active MNV; (**B**) inactive MNV classified as dry AMD; (**C**) active MNV classified as dry AMD; and (**D**) active MNV classified as inactive MNV. Each panel shows matched OCT, outer retina OCTA, and choriocapillaris OCTA images. Cases were selected as the eye with an assigned-class probability closest to the pathway-specific median.

Representative cases from the four most frequent pathways are shown in Figure 6. All four selected eyes had artifact grade 0, indicating that these examples reflected overlapping structural and vascular patterns rather than severe image degradation.

## 4. Discussion

This study evaluated the three-class discrimination of dry age-related macular degeneration (AMD), active macular neovascularization (MNV), and inactive MNV using OCT and OCTA representations extracted with a frozen Vision Transformer. The primary direct multimodal linear SVM achieved an accuracy of 0.753, a macro-F1 score of 0.685, and a macro-AUROC of 0.887. Multimodal fusion provided more balanced performance than direct OCTA fusion, whereas its improvement over OCT alone was not statistically significant. Dry AMD had the highest sensitivity and inactive MNV the lowest. Performance estimates remained similar after grade-3 artifact images were added to the real-world cohort. In addition, most errors were associated with class probabilities near the decision boundary, and occlusion analyses indicated class-dependent reliance on OCT and OCTA.

The advantage of multimodal fusion over OCTA alone, but not over OCT alone, is consistent with current clinical imaging practice. Structural OCT remains the primary modality for assessing neovascular AMD activity because it enables the detection of intraretinal and subretinal fluid and the monitoring of treatment response [1,5,15,16]. OCTA can demonstrate the presence, extent, and vascular morphology of the neovascular complex, but it does not directly depict leakage and is insufficient as a stand-alone basis for treatment decisions [7,8,17,18]. Moreover, the active and inactive MNV reference classes in this study were defined largely according to the presence or absence of fluid on OCT. The strong performance of OCT may therefore partly reflect incorporation of structural OCT findings into the reference standard. Accordingly, the absence of a statistically significant advantage over OCT alone should not be interpreted as evidence that OCTA provides limited biological or clinical information.

The perturbation analyses demonstrated modality-dependent model sensitivity rather than a proven incremental classification benefit from OCTA. OCT occlusion produced the largest overall reduction in predicted-class probability, whereas outer-retina OCTA occlusion more frequently changed the final prediction in active and inactive MNV eyes. Although the occlusion findings indicate sensitivity to OCTA inputs, they do not establish which vascular features influenced the classifier or demonstrate an incremental class-specific benefit over OCT alone. The potential relevance of OCTA near the active–inactive MNV boundary should therefore be considered exploratory. In the primary cohort, eyes with non-exudative MNV were excluded from the dry AMD class; therefore, fewer prediction changes after OCTA occlusion in dry AMD were expected. Nevertheless, whole-modality and patch-occlusion analyses quantify model sensitivity to predefined perturbations and do not establish the causal anatomical importance of specific image regions or identify validated imaging biomarkers.

Previous OCTA-only studies have demonstrated discrimination of AMD activity, although performance decreased across imaging instruments [19,20]. Jin et al. reported 95.5% accuracy in their internal dataset and 100% and 90.48% accuracy in two external datasets for binary active-versus-inactive CNV classification using multimodal OCT–OCTA imaging [21]. Collectively, these findings indicate that OCTA contains information relevant to neovascular activity, but its performance is influenced by image quality, device characteristics, segmentation, class definitions, and validation design. Direct comparison of raw performance estimates is difficult because previous studies generally used binary tasks, different patient spectra, or selected images. In contrast, the present study evaluated dry AMD, active MNV, and inactive MNV within a single three-class task using patient-grouped repeated nested cross-validation.

The main classification challenge was the identification of inactive MNV. In the absence of retinal fluid, the structural OCT appearance of inactive MNV may resemble dry AMD, whereas persistent neovascular flow on OCTA may overlap with active MNV. Anti-VEGF treatment may reduce fine vascular branching while allowing more mature vessels to persist, and slow flow may remain below the OCTA detection threshold [17,18,21]. These overlapping structural and vascular appearances may explain why inactive eyes were classified as either dry AMD or active MNV. The high specificity but low sensitivity for inactive MNV indicates that the model rarely assigned other classes to the inactive category but failed to identify a substantial proportion of truly inactive eyes. Incorporating longitudinal variables—such as treatment burden, time since the last anti-VEGF injection, previous activity patterns, and fibrotic or atrophic lesion characteristics—may improve discrimination of this class. Geographic atrophy status, MNV subtype (types 1–3), and pachychoroid-overlap features were not recorded as structured study variables, limiting detailed disease-stage characterization and phenotype-specific analyses.

The higher performance for dry AMD may reflect the absence of a neovascular complex and the presence of relatively consistent structural findings, including drusen, retinal pigment epithelium alterations, and atrophy. Previous OCT-, OCTA-, and multimodal imaging studies have reported strong discrimination of non-neovascular AMD, automated detection of choroidal neovascularization, and prediction of anti-VEGF treatment indications [11,22,23,24,25]. However, the larger size of the dry AMD group and the lower frequency of severe artifacts in this class may also have contributed to its better classification. Although class weighting and macro-averaged performance metrics were used, unequal class sizes may have contributed to the uneven error distribution, with 40 of 79 misclassifications arising from inactive MNV eyes. However, the influence of class imbalance cannot be separated from the inherent difficulty of distinguishing overlapping structural and vascular phenotypes.

OCTA artifacts can attenuate vascular signals, create false pathological appearances, or obscure the neovascular complex [9,10,26,27,28]. The presence of at least one artifact in 61.7% of eyes reflects a real-world dataset rather than a collection restricted to ideal images. Adding grade-3 artifact images to the real-world cohort did not materially reduce the overall performance estimates. Similarly, the sensitivity analysis in which colored device-generated scan guides were retained produced results broadly consistent with the primary analysis, suggesting limited dependence on this preprocessing choice. However, because the grade-3 artifact cohort was small, these findings should not be interpreted as definitive evidence of robustness to severe artifacts.

In the primary gradable cohort of 320 eyes, more than half of the errors originated from inactive MNV eyes. Assigned-class probabilities for misclassified eyes were generally close to the decision boundary, suggesting that many errors occurred in structurally and vascularly ambiguous cases rather than as highly confident misclassifications. Because grade-3 artifacts were excluded from this analysis, image quality alone cannot explain the observed error pattern. Predictive uncertainty has been associated with error likelihood in medical image classification, and uncertainty-aware approaches may help identify cases requiring expert review [29,30]. Hidden failures within clinically important subgroups nevertheless remain an important safety concern [31]. The model’s probability estimates should therefore be investigated as a means of identifying uncertain cases rather than as a basis for autonomous treatment decisions.

Non-exudative MNV provided a distinct example of disagreement between structural and vascular imaging. In these eyes, structural OCT may resemble dry AMD, whereas OCTA demonstrates neovascular flow, thereby providing conflicting inputs to the three-class model. Of the 13 non-exudative MNV eyes, 61.5% were assigned to dry AMD, while the mean dry AMD probability was only 0.488. Although the sample was too small for definitive conclusions, this finding suggests that non-exudative MNV could be evaluated as a separate fourth class or within a hierarchical framework that first identifies the presence of MNV and subsequently determines exudative activity [32,33,34,35].

In the secondary elastic-net analysis, the direct multimodal model produced higher point estimates than the primary linear SVM for several threshold-dependent metrics, whereas their discrimination estimates were similar. This suggests that the frozen multimodal representation was not entirely dependent on a single downstream linear classifier family. Nevertheless, to preserve the prespecified analysis plan and avoid post hoc model selection based on observed results, the linear SVM remained the primary model for all main and challenge-cohort analyses.

This study has several limitations. Its retrospective, single-center design and the use of a single imaging device limit generalizability to other centers, devices, and patient populations. To reduce internal optimism and information leakage, model development and evaluation used patient-grouped repeated nested cross-validation, with both eyes of a patient retained in the same fold. Standardization, principal component analysis, hyperparameter selection, and probability calibration were performed exclusively within the corresponding training data, and confidence intervals were calculated using patient-clustered bootstrap resampling. These safeguards strengthen internal validity but do not replace independent external validation.

The relatively small inactive MNV, grade-3 artifact, and non-exudative MNV subgroups limited the precision of the corresponding estimates; findings from these challenge cohorts should therefore be considered exploratory. Baseline visual acuity data were unavailable, precluding assessment of their association with fixation-related motion or decentration artifacts. The use of a single horizontal OCT B-scan rather than the complete OCT volume, reliance on automated OCTA slab segmentation, and direct resizing to 224 × 224 pixels may have resulted in information loss or altered spatial proportions. PCA-based dimensionality reduction may have further discarded informative features, and the present approach cannot establish the full potential of multimodal fusion relative to end-to-end deep learning. Requiring complete same-day OCT–OCTA image triplets may have introduced selection bias. Because MNV activity was defined primarily by OCT fluid findings, incorporation bias favoring structural OCT is possible. Although reference diagnoses were jointly established by three ophthalmologists, independent blinded grading was not performed, and intergrader agreement could not be quantified. Furthermore, the reference diagnoses were based on the complete OCT scan set, whereas the classifier received only a single horizontal B-scan. This difference should be considered when interpreting the comparative performance of OCT-only and multimodal models. Although the frozen ImageNet encoder reduced the risk of overfitting, it may also have limited the learning of subtle disease-specific features. The absence of a complete initial screening log prevented reconstruction of pre-inclusion exclusion counts and assessment of consecutive sampling, limiting evaluation of potential selection bias. Finally, the median-intensity inputs used for occlusion were not natural clinical images; therefore, the resulting maps should be interpreted only as measures of model sensitivity to predefined perturbations. Independent calibration, multicenter validation, and clinically defined decision thresholds are required before clinical application.

## 5. Conclusions

Frozen Vision Transformer representations combined with linear classifiers provided a reproducible research framework for three-class discrimination of dry AMD, active MNV, and inactive MNV. Structural OCT carried most of the discriminative information. Although OCTA influenced model predictions in perturbation analyses, multimodal fusion did not significantly outperform OCT alone, and its proposed class-specific contribution remains exploratory. Inactive and non-exudative MNV remained the principal classification challenges. Multicenter external validation across different devices, incorporation of longitudinal clinical variables, and clinically relevant decision thresholds are required before the model can be considered for clinical decision support.

## Figures and Tables

**Table 1:** Cohort characteristics by diagnostic category.

Characteristic	Overall	Dry AMD	Active MNV	Inactive MNV
Eyes, *n*	368	183	105	80
Patients, *n*	200	130	90	69
Age, years	70.3 ± 7.4	69.8 ± 7.5	70.7 ± 7.4	70.9 ± 7.2
Female sex, *n* (%)	151 (41.0)	77 (42.1)	43 (41.0)	31 (38.8)
OCT quality score	86.2 ± 11.0	88.6 ± 10.7	82.1 ± 10.9	86.2 ± 10.1
Any OCTA artifact, *n* (%)	227 (61.7)	101 (55.2)	73 (69.5)	53 (66.2)
Grade-3 artifact, n (%)	35 (9.5)	4 (2.2)	19 (18.1)	12 (15.0)
Non-exudative MNV, *n* (%)	13 (3.5)	13 (7.1)	0	0
Primary cohort, *n* (%)	320 (87.0)	166 (90.7)	86 (81.9)	68 (85.0)

Note: Values are mean ± SD or *n* (%). Age and sex are eye-level. Category-specific patient counts may overlap. AMD = age-related macular degeneration; MNV = macular neovascularization; OCT = optical coherence tomography; OCTA = OCT angiography.

**Table 2:** Primary-cohort performance of the linear SVM models.

Metric	OCT Only	Direct OCTA Fusion	Direct Multimodal Fusion
Accuracy	0.734 (0.688–0.779)	0.694 (0.639–0.745)	0.753 (0.704–0.801)
Balanced accuracy	0.646 (0.594–0.695)	0.602 (0.543–0.655)	0.679 (0.628–0.729)
Macro-F1	0.653 (0.594–0.705)	0.609 (0.544–0.667)	0.685 (0.629–0.737)
MCC	0.554 (0.482–0.620)	0.483 (0.400–0.560)	0.589 (0.518–0.661)
Cohen’s κ	0.543 (0.471–0.610)	0.475 (0.389–0.551)	0.585 (0.513–0.657)
Macro AUROC	0.866 (0.835–0.894)	0.840 (0.802–0.875)	0.887 (0.857–0.914)
Macro AP	0.741 (0.688–0.794)	0.678 (0.626–0.745)	0.740 (0.691–0.800)
Log loss	0.683 (0.631–0.737)	0.766 (0.705–0.827)	0.648 (0.591–0.706)
Brier score	0.383 (0.348–0.421)	0.429 (0.388–0.469)	0.362 (0.322–0.403)
Macro-ECE	0.083 (0.073–0.112)	0.101 (0.087–0.130)	0.096 (0.088–0.124)

Note: Values are estimates (95% patient-clustered bootstrap CIs) based on outer test-fold probabilities averaged across 10 repeated five-fold cross-validation runs. Direct OCTA fusion combined outer retina and choriocapillaris OCTA; direct multimodal fusion additionally included OCT. Higher values indicate better performance except for log loss, Brier score, and macro-ECE. AP, average precision; AUROC, area under the receiver operating characteristic curve; CI, confidence interval; κ, Cohen’s kappa coefficient; macro-ECE, macro-averaged expected calibration error; MCC, Matthews correlation coefficient; OCT, optical coherence tomography; OCTA, optical coherence tomography angiography.

**Table 3:** Class-specific performance of the primary multimodal model.

Class	Sensitivity	Specificity	Precision	F1 Score	AUROC	AP
Dry AMD	0.916 (0.868–0.959)	0.786 (0.714–0.849)	0.822 (0.764–0.876)	0.866 (0.827–0.904)	0.944 (0.916–0.968)	0.955 (0.933–0.975)
Active MNV	0.709 (0.611–0.802)	0.893 (0.852–0.932)	0.709 (0.621–0.805)	0.709 (0.632–0.777)	0.892 (0.852–0.928)	0.758 (0.668–0.841)
Inactive MNV	0.412 (0.300–0.528)	0.917 (0.877–0.952)	0.571 (0.426–0.714)	0.479 (0.364–0.585)	0.824 (0.768–0.875)	0.507 (0.393–0.646)

Note: Values are estimates (95% patient-clustered bootstrap CIs) for the primary linear SVM using direct fusion of OCT, outer retina OCTA, and choriocapillaris OCTA embeddings.

**Table 4:** Performance of the primary multimodal model across multiclass evaluation cohorts.

Cohort	Eyes/Patients, *n*	Accuracy	Balanced Accuracy	Macro-F1	Macro-AUROC
Primary gradable	320/185	0.753 (0.704–0.801)	0.679 (0.628–0.729)	0.685 (0.629–0.737)	0.887 (0.857–0.914)
Real-world	355/198	0.758 (0.715–0.804)	0.699 (0.654–0.745)	0.703 (0.653–0.751)	0.886 (0.859–0.911)
Grade-3 artifact	35/27	0.800 (0.667–0.914)	0.826 (0.730–0.917)	0.832 (0.713–0.919)	0.860 (0.750–0.950)

Note: Values are estimates (95% patient-clustered bootstrap CIs). The primary cohort excluded non-exudative MNV and grade-3 artifacts; the real-world cohort excluded non-exudative MNV but included all artifact grades. Grade-3 cohort estimates are exploratory because of the small sample size.

**Table 5:** Misclassification pathways in the primary cohort.

True Class	Predicted Class	Eyes, *n*	Error Rate, % (95% CI)	Assigned Probability, Median (IQR)
Dry AMD	Active MNV	4	2.4 (0.6–4.9)	0.488 (0.439–0.549)
Dry AMD	Inactive MNV	10	6.0 (2.3–10.4)	0.480 (0.445–0.558)
Active MNV	Dry AMD	14	16.3 (9.0–24.7)	0.549 (0.482–0.637)
Active MNV	Inactive MNV	11	12.8 (5.9–20.0)	0.496 (0.458–0.617)
Inactive MNV	Dry AMD	19	27.9 (17.4–40.0)	0.569 (0.494–0.595)
Inactive MNV	Active MNV	21	30.9 (20.5–42.7)	0.531 (0.473–0.646)

Error rates use the corresponding true-class denominator. Assigned probability is the probability of the selected incorrect class.

## Data Availability

The original contributions presented in this study are included in the article/Supplementary Material. Further inquiries can be directed to the corresponding author.

## Supplementary Materials

The following supporting information can be downloaded at https://www.mdpi.com/article/10.3390/jcm15218647/s1: Supplementary Methods S1–S10: S1: Detailed OCTA Artifact Definitions; S2: Detailed Image Preprocessing, Feature Extraction, and Feature Construction; S3: Patient-Level Split Generation; S4: Exact Model and Hyperparameter Selection Procedures; S5: Calibration and Probability-Based Performance Metrics; S6: Masked Versus Unmasked Preprocessing Comparison; S7: Error and Predictive Uncertainty Analyses; S8: Perturbation-Based Explainability Analysis; S9: Software and Reproducibility Controls; S10: Analytic Cohort Assembly. Supplementary Results S1–S7: S1: Detailed OCTA Artifact Distribution; S2: Hyperparameter Selection and Model-Development Stability; S3: Paired comparison of the primary multimodal SVM with its predefined comparators; S4: Performance of All Predefined Models; S5: Preprocessing Sensitivity Analysis; S6: Perturbation-Based Modality Reliance; S7: Predictive Uncertainty and Local Explanations. Supplementary Tables S1–S7: Table S1: Detailed OCTA artifact distribution by diagnostic category; Table S2: Hyperparameter selection across outer folds; Table S3: Paired comparison of the primary multimodal Linear SVM with the predefined Linear SVM comparators; Table S4: Performance of all predefined models in the primary cohort; Table S5: Masked-versus-unmasked preprocessing sensitivity analysis; Table S6: Whole-modality occlusion results; Table S7: Predictive uncertainty by misclassification pathway. Supplementary Figures S1–S5: Figure S1: Representative examples of OCTA artifact types; Figure S2: Analytic cohort and predefined subgroups; Figure S3: Inner-cross-validation macro-F1 across candidate hyperparameters for the direct multimodal SVM (A) and elastic-net logistic regression (B); Figure S4: Paired masked-versus-unmasked performance differences for the primary multimodal SVM; Figure S5: Modality-specific patch-occlusion maps for the four representative errors shown in main-text Figure 6.
