# Supplementary material for: Multimodal OCT–OCTA Classification of Dry AMD, Active MNV, and Inactive MNV Using Frozen Vision Transformer Features: A Patient-Level Nested Cross-Validation Study
Source: J Clin Med. 2026 Nov 6;15(21):8647. doi: 10.3390/jcm15218647 (PMC13763635; doi:10.3390/jcm15218647)
Supplement: Supplementary file 1 [file jcm-15-08647-s001.zip › jcm-4478632-supplementary.pdf]

Supplementary Methods

Supplementary Method S1. Detailed OCTA Artifact Definitions

Optical coherence tomography angiography (OCTA) images were evaluated for motion or banding, shadowing or masking, segmentation error, projection artifact, blur or defocus, unmasking or window defects, and decentration. More than one artifact type could be assigned to the same eye.

Motion or banding was defined as linear or band-shaped distortion attributable to ocular movement. Shadowing or masking was defined as regional signal attenuation associated with media opacity, hemorrhage, or another obstruction. Segmentation error was defined as inaccurate delineation of slab boundaries resulting in displacement or loss of vascular information. Projection artifact was defined as the appearance of superficial vascular structures within deeper slabs. Blur or defocus represented reduced image sharpness. Unmasking or window defects referred to increased visualization of deeper choroidal structures, particularly in areas of retinal pigment epithelium attenuation. Decentration was defined as substantial displacement of the fovea from the image center or partial exclusion of the fovea from the scan area. Representative examples of the seven OCTA artifact categories are shown in Supplementary Figure S1.

Overall artifact severity was graded according to its effect on lesion discernibility rather than the number of artifact types. Grade 0 indicated no relevant interference with interpretation. Grade 1 indicated that the lesion remained readily discernible. Grade 2 indicated that the lesion could be distinguished only after careful inspection. Grade 3 indicated that lesion assessment was very difficult or clinically unreliable.

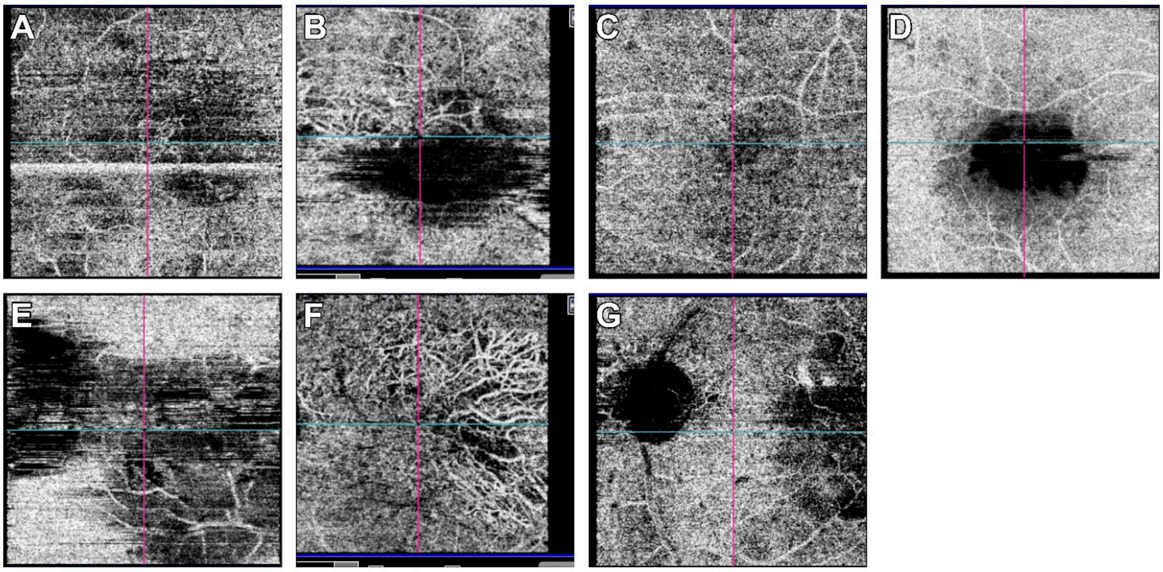

Supplementary Figure S1. Representative examples of OCTA artifact types. (A) motion, **(B)** shadow, **(C)** projection, **(D)** segmentation, **(E)** defocus, **(F)** unmasking, and **(G)** decentration artifacts.

Supplementary Method S2. Detailed Image Preprocessing, Feature Extraction, and Feature Construction

The primary preprocessing strategy was designed to prevent colored device-generated scan guides from becoming spurious classification features. After conversion to red–green–blue color space, a pixel was classified as chromatic when the difference between its maximum and minimum channel values was at least 45 and its maximum channel value was at least 80. Chromatic pixels were deterministically replaced with the corresponding values from a 5×5 grayscale median-filtered version of the image.

The resulting image was converted to grayscale, replicated across three channels, and resized directly to 224×224 pixels using bicubic interpolation. Pixel intensities were scaled to the range of 0–1 and normalized using ImageNet channel means of 0.485, 0.456, and 0.406 and standard deviations of 0.229, 0.224, and 0.225. The same preprocessing procedure was applied independently to the structural OCT, outer retina OCTA, and choriocapillaris OCTA images.

No random rotation, geometric transformation, color modification, blur, or other image augmentation was applied. This provided a deterministic feature-extraction pipeline and avoided augmentation-induced alteration of clinically relevant OCT or OCTA findings.

Features were extracted using a Vision Transformer B/32 model pretrained on ImageNet-1K (torchvision.models.vit_b_32, ViT_B_32_Weights.IMAGENET1K_V1). The original classification head was replaced by an identity mapping, and the model was used in evaluation mode with all weights frozen. No end-to-end fine-tuning was performed.

The same pretrained architecture and weights were applied independently to the three imaging modalities. For each image, the final 768-dimensional class-token representation was retained:

$$f_{OCT}, f_{OR}, f_{CC} \in \mathbb{R}^{768},$$

where OR denotes outer retina OCTA and CC denotes choriocapillaris OCTA.

Embedding extraction was performed once per unique eye because neither the frozen encoder nor the deterministic preprocessing pipeline learned from diagnostic labels, cohort distributions, or cross-validation folds. All embeddings were extracted using 32-bit floating-point precision, with automatic mixed precision disabled.

Three predefined feature configurations were evaluated using the primary classifier family. The OCT-only configuration contained the 768-dimensional structural OCT embedding. Direct OCTA fusion was constructed by concatenating the outer retina and choriocapillaris embeddings:

$$f_{OCTA} = f_{OR} \oplus f_{CC} \in \mathbb{R}^{1536}.$$

Direct multimodal fusion was constructed by concatenating all three modality-specific embeddings:

$$f_{MM} = f_{OCT} \oplus f_{OR} \oplus f_{CC} \in \mathbb{R}^{2304}.$$

Direct multimodal fusion was prespecified as the primary input configuration. Feature concatenation did not involve learned gating, feature weighting, or quality and artifact metadata. Age, sex, OCT image-quality score, artifact type, and artifact severity were used only for cohort definition, descriptive analysis, fold balancing, and predefined challenge analyses and were not supplied to the classifiers.

Supplementary Method S3. Patient-Level Split Generation

Repeated nested cross-validation splits were generated before embedding extraction and model development. The validation framework comprised 10 repetitions, five outer folds, and four inner folds. Assignment was performed at the patient level so that all eligible eyes from the same patient remained within the same fold.

For each repetition, every patient served in an outer-test fold exactly once. Within each outer-training set, the four patient-grouped inner folds were used to fit feature standardization and principal component analysis (PCA), select hyperparameters, and, where applicable, calibrate class probabilities. Outer-test observations did not contribute to transformation fitting, model fitting, hyperparameter selection, or calibration.

Patient-level variables used solely for fold balancing included the number of available eyes, the number and diagnostic distribution of primary and real-world eyes, artifact-severity counts, the number of non-exudative macular neovascularization eyes, the presence of any OCTA artifact, sex, age quartile, and one-eye versus two-eye participation. None of these balancing variables was provided to the classifiers.

A deterministic multi-start greedy assignment followed by pairwise local improvement minimized weighted deviations from the target fold distributions while constraining patient counts across folds to differ by no more than one. Split selection was independent of subsequent model performance.

Integrity checks confirmed that both eyes from the same patient remained together, no outer-test patient entered an inner fold, and all three primary diagnostic classes were represented in every outer and inner validation fold.

Supplementary Method S4. Exact Model and Hyperparameter Selection Procedures

Before classifier fitting, features were standardized using StandardScaler and reduced using principal component analysis (PCA). Standardization and PCA were refitted within every inner-training fold and applied to the corresponding inner-validation fold. PCA used randomized singular-value decomposition without whitening and retained up to 128 components. The

effective number of components was reduced when required by the number of available training observations or input features.

A class-weighted linear support vector machine (SVM) was prespecified as the primary classifier. The model used an L2 penalty, squared-hinge loss, and balanced class weights. Candidate regularization parameters were:

$C \in \{0.01, 0.1, 1, 10\}$.

For each outer-training set, candidate models generated pooled inner out-of-fold predictions. Candidates were ranked primarily by pooled macro-F1, followed by balanced accuracy, lower between-fold macro-F1 variability, and stronger regularization when preceding criteria were tied. After hyperparameter selection, the transformations and classifier were refitted using the complete outer-training set.

Elastic-net multinomial logistic regression was evaluated as a predefined secondary classifier. It used the SAGA solver, balanced class weights, regularization parameters of:

$C \in \{0.01, 0.1, 1\}$,

and elastic-net mixing parameters of:

$l_1 ratio \in \{0.25, 0.75\}$.

Secondary input configurations included OCT alone, outer retina OCTA alone, choriocapillaris OCTA alone, direct OCTA fusion, and direct multimodal fusion. Candidate models were ranked using pooled inner out-of-fold macro-F1, with subsequent tie-breaking based on balanced accuracy, lower log loss, and the prespecified preference for the more parsimonious solution.

Because the pretrained encoder remained frozen and the downstream estimators were convex linear classifiers, model development did not involve neural-network epochs or end-to-end training and validation loss curves. Model-development behavior was instead summarized using pooled inner out-of-fold macro-F1 across the prespecified hyperparameter grid within each of the 50 outer model-development iterations generated by 10 repetitions of five-fold outer cross-validation.

Supplementary Method S5. Calibration and Probability-Based Performance Metrics

Following hyperparameter selection, linear SVM decision scores were converted to class probabilities using sigmoid calibration. The four patient-grouped inner folds from the corresponding outer-training set were reused as calibration folds. This ensured that eyes from the same patient were not divided between calibration-training and calibration-validation subsets.

Probabilities were aligned to the fixed class order of dry age-related macular degeneration, active macular neovascularization, and inactive macular neovascularization and were renormalized to sum to one when required. Each eye received one outer out-of-fold probability vector in each of

the 10 repetitions. Final eye-level probabilities were calculated by averaging these 10 vectors, and the class with the highest mean probability was selected as the final prediction.

Average precision was calculated using average_precision_score and is therefore reported as average precision rather than as a trapezoidally integrated area under the precision–recall curve.

The multiclass Brier score was calculated as the mean sum of squared differences between the three predicted class probabilities and the corresponding one-hot encoded reference labels. Expected calibration error was calculated separately for each class using a one-versus-rest formulation and 10 equal-width probability bins. Empty bins made no contribution to the expected calibration error sum. Macro expected calibration error was calculated as the unweighted mean of the three class-specific values.

Supplementary Method S6. Masked Versus Unmasked Preprocessing Comparison

A predefined sensitivity analysis examined whether removing the colored scan-guide masking step materially affected the performance of the prespecified direct multimodal linear SVM. In the unmasked preprocessing variant, chromatic overlays were retained before grayscale conversion. All subsequent resizing, intensity scaling, ImageNet normalization, and frozen feature-extraction procedures remained unchanged.

The masked and unmasked analyses used identical patient-level repeated nested cross-validation splits, frozen feature extractors, feature configurations, standardization procedures, PCA specifications, classifier families, and probability-calibration strategies. Hyperparameters were selected independently within each unmasked inner cross-validation loop.

Masked and unmasked eye-level probabilities were compared using paired patient-clustered bootstrap resampling. For higher-is-better metrics, paired differences were calculated as masked minus unmasked performance. For error and calibration metrics, differences were calculated as unmasked minus masked performance so that positive values consistently favored the primary masked preprocessing. The same patient-bootstrap samples were used for both preprocessing variants.

Supplementary Method S7. Error and Predictive Uncertainty Analyses

All six possible off-diagonal misclassification pathways were quantified in the primary gradable cohort. For each pathway, the number of misclassified eyes and the error rate within the corresponding true diagnostic class were calculated. Confidence intervals for pathway-specific error rates were estimated using 2000 patient-clustered bootstrap replicates.

For each misclassified eye, the assigned-class probability was defined as the mean outer out-of-fold probability of the class selected by the final model after averaging across the 10 repetitions. Across-repeat uncertainty was summarized using the standard deviation of the assigned-class probability across the 10 outer-test predictions.

Predictive entropy was calculated from the averaged three-class probability vector:

$H(p) = -\sum_{c=1}^{3} p_c \log(p_c)$,

where p_c denotes the predicted probability of class c. Higher entropy indicates a less concentrated probability distribution and therefore greater predictive uncertainty.

One representative eye was selected from each of the four most frequent misclassification pathways. Within each pathway, the representative eye was selected deterministically as the observation whose assigned-class probability was closest to the pathway-specific median. Different patients were used across the four representative examples. Case selection was completed before the perturbation-based explainability analysis.

Supplementary Method S8. Perturbation-Based Explainability Analysis

Perturbation-based analyses were performed using the exact patient-disjoint outer out-of-fold models assigned to each eye. Global whole-modality occlusion was assessed in all eyes in the primary gradable cohort. For each analysis, the complete *224 × 224* input of one modality was replaced with that image's median grayscale intensity while the other two modality embeddings remained unchanged. The perturbed image was re-encoded using the frozen ViT-B/32 encoder and evaluated using the corresponding calibrated linear SVM.

Changes in model-predicted and reference-class probabilities were averaged across the 10 repeated outer out-of-fold models. Prediction flip rate was defined as the proportion of eyes for which the final averaged predicted class changed after occlusion. Mean probability changes and prediction flip rates were summarized using patient-clustered bootstrap confidence intervals.

Local patch-occlusion maps were generated for the four representative misclassification cases selected in Supplementary Method S7. Within each modality, a *32 × 32*-pixel region was sequentially replaced with the image-specific median grayscale intensity using a 16-pixel stride. Each perturbed image was re-encoded, combined with the unchanged embeddings of the other two modalities, and evaluated using the corresponding calibrated outer out-of-fold SVM.

Signed occlusion importance was defined as the original model-predicted class probability minus the probability obtained after occlusion and was averaged across the 10 repeated outer out-of-fold models. Positive values indicated regions whose removal reduced the predicted-class probability and therefore supported the original model decision. Negative values indicated regions whose removal increased that probability.

Whole-modality and patch-occlusion analyses quantify model sensitivity to predefined, nonphysiological image perturbations. They should not be interpreted as demonstrating causal anatomical importance or as identifying validated imaging biomarkers.

Supplementary Method S9. Software and Reproducibility Controls

Analyses were performed using Python 3.12.10. Frozen feature extraction used PyTorch 2.5.1 and torchvision 0.20.1. Model development and evaluation used scikit-learn 1.7.2, NumPy 2.3.5, and pandas 3.0.0. The global random seed was fixed at 20260715.

The pretrained model checkpoint, source images, extracted embeddings, patient-level split manifests, analysis scripts, and principal output files were verified using SHA-256 hashes. Deterministic PyTorch algorithms were enabled, cuDNN benchmarking was disabled, and both TensorFloat-32 and automatic mixed precision were disabled.

Intermediate checkpoints, selected hyperparameters, model-fitting diagnostics, package versions, execution commands, and integrity-control results were retained to support reproducibility and auditability.

Supplementary Method S10. Analytic Cohort Assembly

The final analytical dataset comprised 368 eyes from 200 patients. Cohort allocation was based on diagnostic category and OCTA artifact severity, as illustrated in Supplementary Figure S2. Subgroups were mutually exclusive at the eye level, although patient membership could overlap.

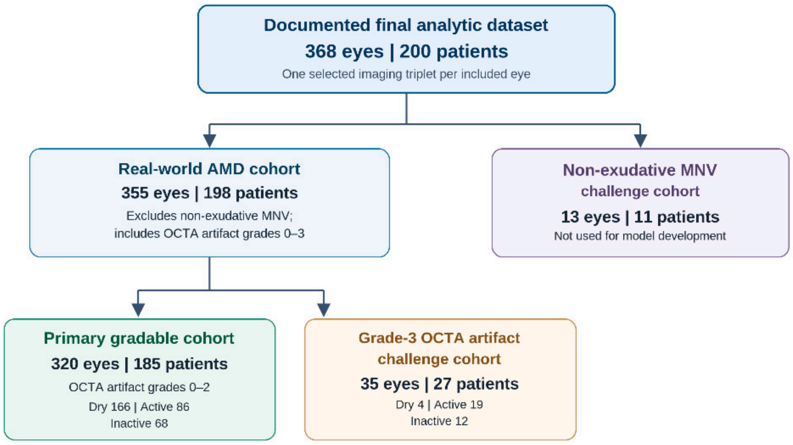

Figure S2. Analytic cohort and predefined subgroups. The final dataset comprised 368 eyes from 200 patients. The real-world cohort included 355 eyes from 198 patients, comprising the primary gradable cohort (320 eyes, 185 patients) and grade-3 artifact cohort (35 eyes, 27 patients). Non-exudative MNV was evaluated separately (13 eyes, 11 patients).

Supplementary Results

Supplementary Results S1. Detailed OCTA Artifact Distribution

OCTA artifacts were more frequent in active and inactive MNV than in dry AMD. Motion, shadowing, and segmentation errors were concentrated in the MNV groups, whereas projection artifacts were common across all diagnostic categories (Supplementary Table S1).

Supplementary Table S1. Detailed OCTA artifact distribution by diagnostic category

**Characteristic, *n* (%)**	**Overall**	**Dry AMD**	**Active MNV**	**Inactive MNV**
Any artifact	227 (61.7)	101 (55.2)	73 (69.5)	53 (66.2)
Severity 0	235 (63.9)	143 (78.1)	48 (45.7)	44 (55.0)
Severity 1	56 (15.2)	22 (12.0)	21 (20.0)	13 (16.2)
Severity 2	42 (11.4)	14 (7.7)	17 (16.2)	11 (13.8)
Severity 3	35 (9.5)	4 (2.2)	19 (18.1)	12 (15.0)
Motion	96 (26.1)	22 (12.0)	48 (45.7)	26 (32.5)
Shadow	70 (19.0)	17 (9.3)	40 (38.1)	13 (16.2)
Segmentation	46 (12.5)	9 (4.9)	22 (21.0)	15 (18.8)
Projection	119 (32.3)	65 (35.5)	35 (33.3)	19 (23.8)
Blur	34 (9.2)	10 (5.5)	14 (13.3)	10 (12.5)
Unmasking	18 (4.9)	11 (6.0)	2 (1.9)	5 (6.2)
Decentration	39 (10.6)	11 (6.0)	11 (10.5)	17 (21.2)

Note: Artifact types were not mutually exclusive. AMD = age-related macular degeneration; MNV = macular neovascularization; OCTA = OCT angiography.

Supplementary Results S2. Hyperparameter Selection and Model-Development Stability

Stronger regularization was generally preferred by both classifier families. Selection frequencies and corresponding inner-cross-validation performance are summarized in Supplementary Table S2.

Supplementary Table S2. Hyperparameter selection across outer folds

**Model/input**	**Selected C, *n***	**Selected l1 ratio, *n***	**Inner-CV macro-F1**
SVM/OCT	42/7/1/0	—	0.644 ± 0.030
SVM/OCTA	37/9/2/2	—	0.627 ± 0.027
SVM/OCT + OCTA	38/9/2/1	—	0.679 ± 0.027
Elastic net/OCT	8/28/14	28/22	0.649 ± 0.022
Elastic net/OR-OCTA	24/24/2	32/18	0.614 ± 0.024
Elastic net/CC-OCTA	35/10/5	39/11	0.606 ± 0.021
Elastic net/OCTA	33/12/5	36/14	0.662 ± 0.024
Elastic net/OCT + OCTA	35/10/5	36/14	0.704 ± 0.018

Note: C counts are ordered as 0.01/0.1/1/10 for SVM and 0.01/0.1/1 for elastic net; l1-ratio counts are ordered as 0.25/0.75. Values are selections across 50 outer folds. CC = choriocapillaris; OR = outer retina; SVM = support vector machine.

Inner-cross-validation performance varied only modestly across candidate hyperparameters, supporting the stability of the selection procedure rather than indicating a sharply defined optimum (Supplementary Figure S3).

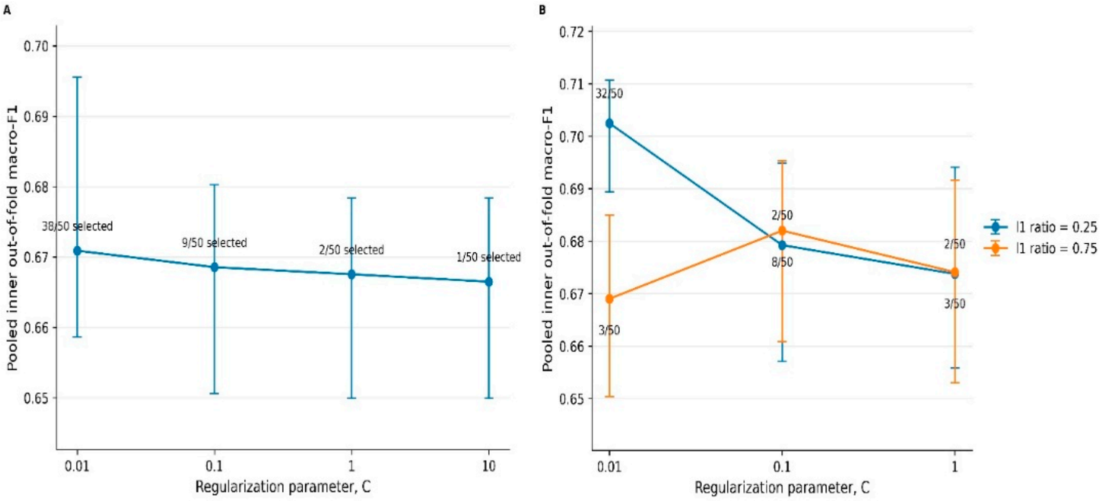

Supplementary Figure S3. Inner-cross-validation macro-F1 across candidate hyperparameters for the direct multimodal SVM (A) and elastic-net logistic regression (B). Points and error bars show the median and interquartile range across 50 outer model-development iterations; labels indicate selection counts. SVM = support vector machine.

For the Linear SVM, the median pooled inner out-of-fold macro-F1 ranged from approximately 0.666 to 0.671 across the candidate *C* values. The elastic-net model showed its highest median inner macro-F1 with stronger regularization and the lower l1 ratio. These results support the stability of the nested model-selection process rather than indicating a sharply defined optimum (Supplementary Figure S3).

Supplementary Results S3. Paired comparison of the primary multimodal SVM with its predefined comparators

Direct multimodal fusion consistently outperformed direct OCTA fusion. In contrast, paired differences between multimodal fusion and OCT alone were small and statistically nonsignificant (Supplementary Table S3).

Supplementary Table S3. Paired comparison of the primary multimodal Linear SVM with the predefined Linear SVM comparators

**Metric**	**Multimodal vs. OCT, Δ (95% CI)**	**Adjusted *p***	**Multimodal vs OCTA, Δ (95% CI)**	**Adjusted *p***
Macro-F1	0.032 (−0.027–0.090)	0.288	0.075 (0.020–0.133)	0.012
BAcc	0.033 (−0.021–0.086)	0.220	0.077 (0.027–0.132)	0.004
Macro-AUROC	0.020 (−0.008–0.048)	0.138	0.047 (0.026–0.068)	<0.001
Macro-AP	−0.001 (−0.045–0.052)	0.948	0.062 (0.023–0.098)	0.002
MCC	0.035 (−0.045–0.116)	0.407	0.106 (0.037–0.175)	0.002

Note: Δ = multimodal minus comparator. Holm-adjusted p values were obtained by paired patient-clustered bootstrap. AP = average precision; AUROC = area under the receiver operating characteristic curve; BAcc = balanced accuracy; MCC = Matthews correlation coefficient.

Supplementary Results S4. Performance of All Predefined Models

Across both classifier families, combining OCT with OCTA produced more balanced performance than OCTA alone. The multimodal elastic-net model had the highest point estimates for several threshold-dependent metrics, whereas multimodal AUROC estimates were similar between classifier families (Supplementary Table S4).

Supplementary Table S4. Performance of all predefined models in the primary cohort

Panel A. Classification performance

Model/input	Accuracy	BAcc	Macro-F1	MCC	Cohen's κ
SVM/OCT	0.734 (0.688–0.779)	0.646 (0.594–0.695)	0.653 (0.594–0.705)	0.554 (0.482–0.620)	0.543 (0.471–0.610)
SVM/OCTA	0.694 (0.639–0.745)	0.602 (0.543–0.655)	0.609 (0.544–0.667)	0.483 (0.400–0.560)	0.475 (0.389–0.551)
SVM/OCT + OCTA	0.753 (0.704–0.801)	0.679 (0.628–0.729)	0.685 (0.629–0.737)	0.589 (0.518–0.661)	0.585 (0.513–0.657)
Elastic net/OCT	0.725 (0.677–0.774)	0.676 (0.621–0.730)	0.676 (0.621–0.728)	0.552 (0.478–0.628)	0.552 (0.477–0.627)
Elastic net/OR-OCTA	0.659 (0.606–0.712)	0.612 (0.554–0.670)	0.609 (0.551–0.664)	0.451 (0.370–0.531)	0.450 (0.370–0.530)
Elastic net/CC-OCTA	0.662 (0.605–0.715)	0.610 (0.546–0.670)	0.609 (0.544–0.667)	0.453 (0.368–0.535)	0.453 (0.366–0.534)
Elastic net/OCTA	0.725 (0.669–0.776)	0.678 (0.617–0.735)	0.675 (0.614–0.731)	0.555 (0.472–0.633)	0.555 (0.471–0.632)
Elastic net/OCT + OCTA	0.775 (0.726–0.822)	0.730 (0.672–0.784)	0.729 (0.672–0.779)	0.635 (0.560–0.706)	0.634 (0.558–0.704)

Panel B. Discrimination and probabilistic performance

Model/input	Macro-AUROC	Macro-AP	Log loss	Brier	Macro-ECE
SVM/OCT	0.866 (0.835–0.894)	0.741 (0.688–0.794)	0.683 (0.631–0.737)	0.383 (0.348–0.421)	0.083 (0.073–0.112)
SVM/OCTA	0.840 (0.802–0.875)	0.678 (0.626–0.745)	0.766 (0.705–0.827)	0.429 (0.388–0.469)	0.101 (0.087–0.130)
SVM/OCT + OCTA	0.887 (0.857–0.914)	0.740 (0.691–0.800)	0.648 (0.591–0.706)	0.362 (0.322–0.403)	0.096 (0.088–0.124)
Elastic net/OCT	0.852 (0.819–0.884)	0.725 (0.670–0.781)	0.699 (0.609–0.792)	0.396 (0.342–0.452)	0.059 (0.056–0.095)

Elastic net/OR-OCTA	0.819 (0.778–0.857)	0.669 (0.613–0.736)	0.782 (0.702–0.873)	0.442 (0.393–0.493)	0.066 (0.061–0.106)
Elastic net/CC-OCTA	0.806 (0.766–0.846)	0.631 (0.580–0.699)	0.815 (0.744–0.893)	0.465 (0.421–0.512)	0.080 (0.078–0.117)
Elastic net/OCTA	0.850 (0.810–0.884)	0.699 (0.643–0.763)	0.722 (0.640–0.812)	0.396 (0.350–0.448)	0.081 (0.074–0.114)
Elastic net/OCT + OCTA	0.886 (0.852–0.917)	0.750 (0.696–0.815)	0.613 (0.537–0.697)	0.342 (0.294–0.396)	0.062 (0.060–0.096)

Note: Values are estimates (95% patient-clustered bootstrap CIs). AP = average precision; BAcc = balanced accuracy; ECE = expected calibration error; MCC = Matthews correlation coefficient; SVM = support vector machine.

Supplementary Results S5. Preprocessing Sensitivity Analysis

Removing chromatic scan-guide masking produced only small differences in the primary and real-world cohorts. Larger differences in the grade-3 cohort were imprecise because of the limited sample size (Supplementary Table S5).

Supplementary Table S5. Masked-versus-unmasked preprocessing sensitivity analysis

Panel A. Primary cohort

Metric	Masked	Unmasked	Δ favoring masked
Accuracy	0.753 (0.704–0.801)	0.741 (0.688–0.789)	0.013 (−0.013 to 0.038)
BAcc	0.679 (0.628–0.729)	0.653 (0.600–0.704)	0.026 (−0.008 to 0.062)
Macro-F1	0.685 (0.629–0.737)	0.660 (0.601–0.716)	0.025 (−0.015 to 0.067)
MCC	0.589 (0.518–0.661)	0.565 (0.488–0.642)	0.024 (−0.020 to 0.068)
Cohen’s κ	0.585 (0.513–0.657)	0.556 (0.476–0.631)	0.030 (−0.013 to 0.075)
Macro AUROC	0.887 (0.857–0.914)	0.893 (0.865–0.920)	−0.006 (−0.017 to 0.005)
Macro AP	0.740 (0.691–0.800)	0.746 (0.697–0.808)	−0.006 (−0.029 to 0.016)
Log loss	0.648 (0.591–0.706)	0.638 (0.585–0.694)	−0.009 (−0.027 to 0.009)
Brier	0.362 (0.322–0.403)	0.360 (0.321–0.401)	−0.002 (−0.014 to 0.009)
Macro-ECE	0.096 (0.088–0.124)	0.096 (0.089–0.125)	−0.000 (−0.016 to 0.017)

Panel B. Real-world cohort

Metric	Masked	Unmasked	Δ favoring masked
Accuracy	0.758 (0.715–0.804)	0.738 (0.690–0.784)	0.020 (−0.006 to 0.043)
BAcc	0.699 (0.654–0.745)	0.668 (0.621–0.716)	0.032 (−0.000 to 0.062)
Macro-F1	0.703 (0.653–0.751)	0.671 (0.619–0.723)	0.032 (−0.005 to 0.066)
MCC	0.612 (0.545–0.677)	0.578 (0.509–0.649)	0.033 (−0.007 to 0.071)
Cohen's κ	0.608 (0.541–0.674)	0.570 (0.499–0.641)	0.038 (−0.003 to 0.077)
Macro-AUROC	0.886 (0.859–0.911)	0.889 (0.862–0.913)	−0.003 (−0.014 to 0.007)
Macro-AP	0.751 (0.707–0.806)	0.751 (0.704–0.806)	0.000 (−0.019 to 0.019)
Log loss	0.657 (0.600–0.713)	0.652 (0.599–0.707)	−0.005 (−0.022 to 0.011)
Brier	0.367 (0.331–0.403)	0.368 (0.331–0.407)	0.001 (−0.011 to 0.012)
Macro-ECE	0.093 (0.086–0.119)	0.095 (0.087–0.122)	0.003 (−0.016 to 0.016)

Panel C. Grade-3 artifact cohort

Metric	Masked	Unmasked	Δ favoring masked
Accuracy	0.800 (0.667–0.914)	0.714 (0.576–0.848)	0.086 (0.000–0.184)
BAcc	0.826 (0.730–0.917)	0.763 (0.662–0.860)	0.063 (0.000–0.143)
Macro-F1	0.832 (0.713–0.919)	0.735 (0.582–0.845)	0.097 (0.000–0.250)
MCC	0.650 (0.434–0.855)	0.506 (0.253–0.736)	0.145 (0.000–0.314)
Cohen's κ	0.641 (0.406–0.846)	0.504 (0.248–0.726)	0.138 (0.000–0.299)
Macro AUROC	0.860 (0.750–0.950)	0.837 (0.730–0.931)	0.023 (−0.008 to 0.053)
Macro AP	0.852 (0.761–0.947)	0.802 (0.715–0.920)	0.050 (−0.007 to 0.086)
Log loss	0.740 (0.594–0.910)	0.772 (0.632–0.919)	0.032 (−0.016 to 0.081)
Multiclass Brier score	0.415 (0.325–0.510)	0.443 (0.352–0.536)	0.028 (−0.002 to 0.058)
Macro-ECE	0.165 (0.151–0.247)	0.147 (0.147–0.252)	−0.018 (−0.049 to 0.039)

Note: Values are estimates (95% patient-clustered bootstrap CIs). Δ was oriented so that positive values favor masking. AP = average precision; BAcc = balanced accuracy; ECE = expected calibration error; MCC = Matthews correlation coefficient.

Paired differences were generally centered near zero in the primary cohort, indicating limited dependence of the principal findings on the masking procedure (Supplementary Figure ~~S2~~ S4).

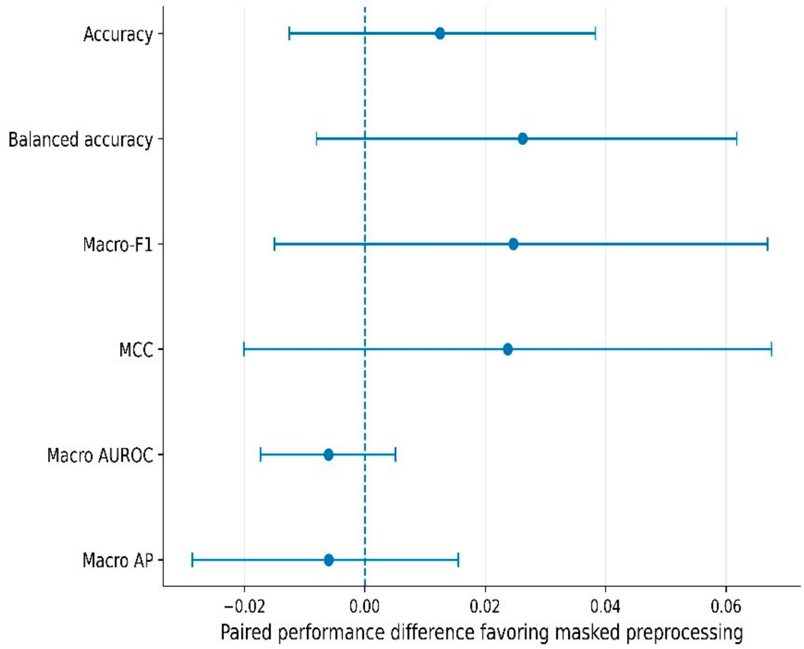

Supplementary Figure S4 . Paired masked-versus-unmasked performance differences for the primary multimodal SVM. Points and error bars show differences and 95% patient-clustered bootstrap confidence intervals. Positive values favor masking. AP = average precision; AUROC = area under the receiver operating characteristic curve; MCC = Matthews correlation coefficient; SVM = support vector machine.

Supplementary Results S6. Perturbation-Based Modality Reliance

Whole-modality occlusion showed class-dependent reliance on the three imaging inputs. OCT removal produced the largest mean probability decrease overall, whereas outer retina OCTA removal produced the highest prediction flip rate (Supplementary Table S6).

Supplementary Table S6. Whole-modality occlusion results

Panel A. All primary-cohort eyes (320 eyes/185 patients)

Occluded modality	Predicted-class Δp	Reference-class Δp	Flip rate, %
OCT	0.132 (0.118–0.146)	0.106 (0.090–0.122)	16.6 (12.5–20.9)
OR-OCTA	0.084 (0.072–0.096)	0.057 (0.041–0.072)	24.4 (19.8–29.1)
CC-OCTA	0.060 (0.053–0.068)	0.036 (0.026–0.046)	12.8 (9.2–16.9)

Panel B. Dry AMD (166 eyes/123 patients)

Occluded modality	Predicted-class Δp	Reference-class Δp	Flip rate, %
OCT	0.172 (0.155–0.189)	0.183 (0.167–0.198)	7.2 (3.6–11.2)
OR-OCTA	0.046 (0.034–0.059)	0.002 (−0.019–0.020)	7.2 (3.1–12.0)
CC-OCTA	0.061 (0.053–0.068)	0.045 (0.032–0.057)	3.6 (1.2–6.6)

Panel C. Active MNV (86 eyes/77 patients)

Occluded modality	Predicted-class Δp	Reference-class Δp	Flip rate, %
OCT	0.107 (0.084–0.131)	0.086 (0.059–0.112)	24.4 (15.3–33.7)
OR-OCTA	0.123 (0.098–0.149)	0.130 (0.106–0.154)	32.6 (23.3–42.9)
CC-OCTA	0.067 (0.047–0.089)	0.043 (0.017–0.068)	20.9 (12.9–29.8)

Panel D. Inactive MNV (68 eyes/59 patients)

Occluded modality	Predicted-class Δp	Reference-class Δp	Flip rate, %
OCT	0.066 (0.036–0.099)	−0.057 (−0.079–−0.036)	29.4 (19.1–40.3)
OR-OCTA	0.128 (0.095–0.161)	0.100 (0.072–0.128)	55.9 (43.9–67.6)
CC-OCTA	0.052 (0.034–0.071)	0.006 (−0.009–0.022)	25.0 (14.1–36.8)

Note: Values are means or percentages (95% patient-clustered bootstrap CI). Positive Δp indicates a probability decrease after occlusion. CC = choriocapillaris; OR = outer retina.

Supplementary Results S7. Predictive Uncertainty and Local Explanations

Assigned-class probabilities, predictive entropy, and across-repeat variability for each misclassification pathway are summarized in Supplementary Table S7. Uncertainty was generally high, with assigned probabilities frequently close to the decision boundary.

Supplementary Table S7. Predictive uncertainty by misclassification pathway

Pathway	Eyes, *n*	Assigned p, median (IQR)	Entropy, median (IQR)	Repeat SD, median (IQR)
Dry AMD → Active MNV	4	0.488 (0.439–0.549)	1.043 (0.995–1.065)	0.073 (0.060–0.083)
Dry AMD → Inactive MNV	10	0.480 (0.445–0.558)	1.031 (0.992–1.064)	0.074 (0.069–0.082)
Active MNV → Dry AMD	14	0.549 (0.482–0.637)	0.929 (0.880–0.991)	0.062 (0.046–0.072)
Active MNV → Inactive MNV	11	0.496 (0.458–0.617)	1.010 (0.818–1.031)	0.071 (0.055–0.092)
Inactive MNV → Dry AMD	19	0.569 (0.494–0.595)	0.964 (0.911–1.020)	0.060 (0.049–0.072)
Inactive MNV → Active MNV	21	0.531 (0.473–0.646)	0.925 (0.817–1.020)	0.064 (0.050–0.072)

Note: Assigned *p* is the probability of the selected incorrect class. Higher entropy indicates greater uncertainty. AMD = age-related macular degeneration; IQR = interquartile range; MNV = macular neovascularization; SD = standard deviation.

Local occlusion maps for the same four representative cases shown in main-text Figure 6 demonstrated heterogeneous modality- and region-specific sensitivity (Supplementary Figure S5).

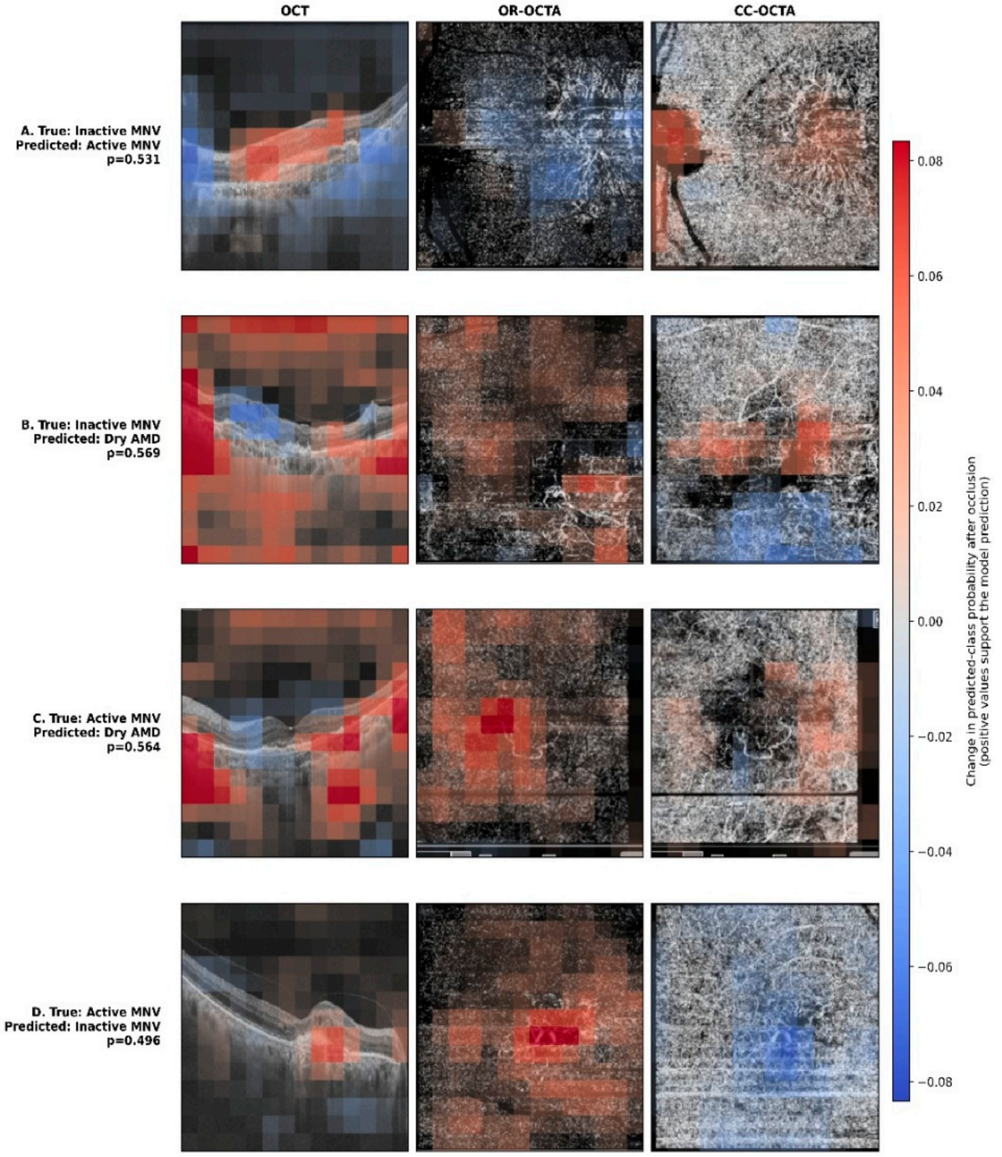

Supplementary Figure S5. Modality-specific patch-occlusion maps for the four representative errors shown in main-text Figure 6. Each row shows OCT, outer retina OCTA, and choriocapillaris OCTA inputs and their corresponding sensitivity maps. Positive values indicate regions whose occlusion reduced the predicted-class probability. These maps represent sensitivity to predefined perturbations rather than causal anatomical importance. MNV = macular neovascularization; OCT = optical coherence tomography; OCTA = OCT angiography.
